# Metabolic engineering of *Escherichia coli* into a versatile glycosylation platform: production of bio-active quercetin glycosides

**DOI:** 10.1186/s12934-015-0326-1

**Published:** 2015-09-16

**Authors:** Frederik De Bruyn, Maarten Van Brempt, Jo Maertens, Wouter Van Bellegem, Dries Duchi, Marjan De Mey

**Affiliations:** Department of Biochemical and Microbial Technology, Centre of Expertise-Industrial Biotechnology and Biocatalysis, Ghent University, Coupure Links 653, 9000 Ghent, Belgium

**Keywords:** Galactosylation, Rhamnosylation, Glycosylation, Hyperoside, Quercitrin, *Escherichia coli* W, Metabolic engineering, Flavonoids

## Abstract

**Background:**

Flavonoids are bio-active specialized plant metabolites which mainly occur as different glycosides. Due to the increasing market demand, various biotechnological approaches have been developed which use *Escherichia coli* as a microbial catalyst for the stereospecific glycosylation of flavonoids. Despite these efforts, most processes still display low production rates and titers, which render them unsuitable for large-scale applications.

**Results:**

In this contribution, we expanded a previously developed in vivo glucosylation platform in *E. coli* W, into an efficient system for selective galactosylation and rhamnosylation. The rational of the novel metabolic engineering strategy constitutes of the introduction of an alternative sucrose metabolism in the form of a sucrose phosphorylase, which cleaves sucrose into fructose and glucose 1-phosphate as precursor for UDP-glucose. To preserve these intermediates for glycosylation purposes, metabolization reactions were knocked-out. Due to the pivotal role of UDP-glucose, overexpression of the interconverting enzymes *galE* and *MUM4* ensured the formation of both UDP-galactose and UDP-rhamnose, respectively. By additionally supplying exogenously fed quercetin and overexpressing a flavonol galactosyltransferase (F3GT) or a rhamnosyltransferase (RhaGT), 0.94 g/L hyperoside (quercetin 3-*O*-galactoside) and 1.12 g/L quercitrin (quercetin 3-*O*-rhamnoside) could be produced, respectively. In addition, both strains showed activity towards other promising dietary flavonols like kaempferol, fisetin, morin and myricetin.

**Conclusions:**

Two *E. coli* W mutants were engineered that could effectively produce the bio-active flavonol glycosides hyperoside and quercitrin starting from the cheap substrates sucrose and quercetin. This novel fermentation-based glycosylation strategy will allow the economically viable production of various glycosides.

**Electronic supplementary material:**

The online version of this article (doi:10.1186/s12934-015-0326-1) contains supplementary material, which is available to authorized users.

## Background

Flavonoids are a class of plant secondary metabolites, which are chemically characterized by a 15-carbon backbone that consists of two phenyl rings and a heterocyclic ring. To date, over 10,000 flavonoids have been characterized from various plants, which are classified according to their chemical structure, i.e., the number and presence of hydroxyl groups and further functional group modifications into various subgroups, such as anthoxanthins, flavanones, and flavanonols [1, 2].

**Fig. 1:**
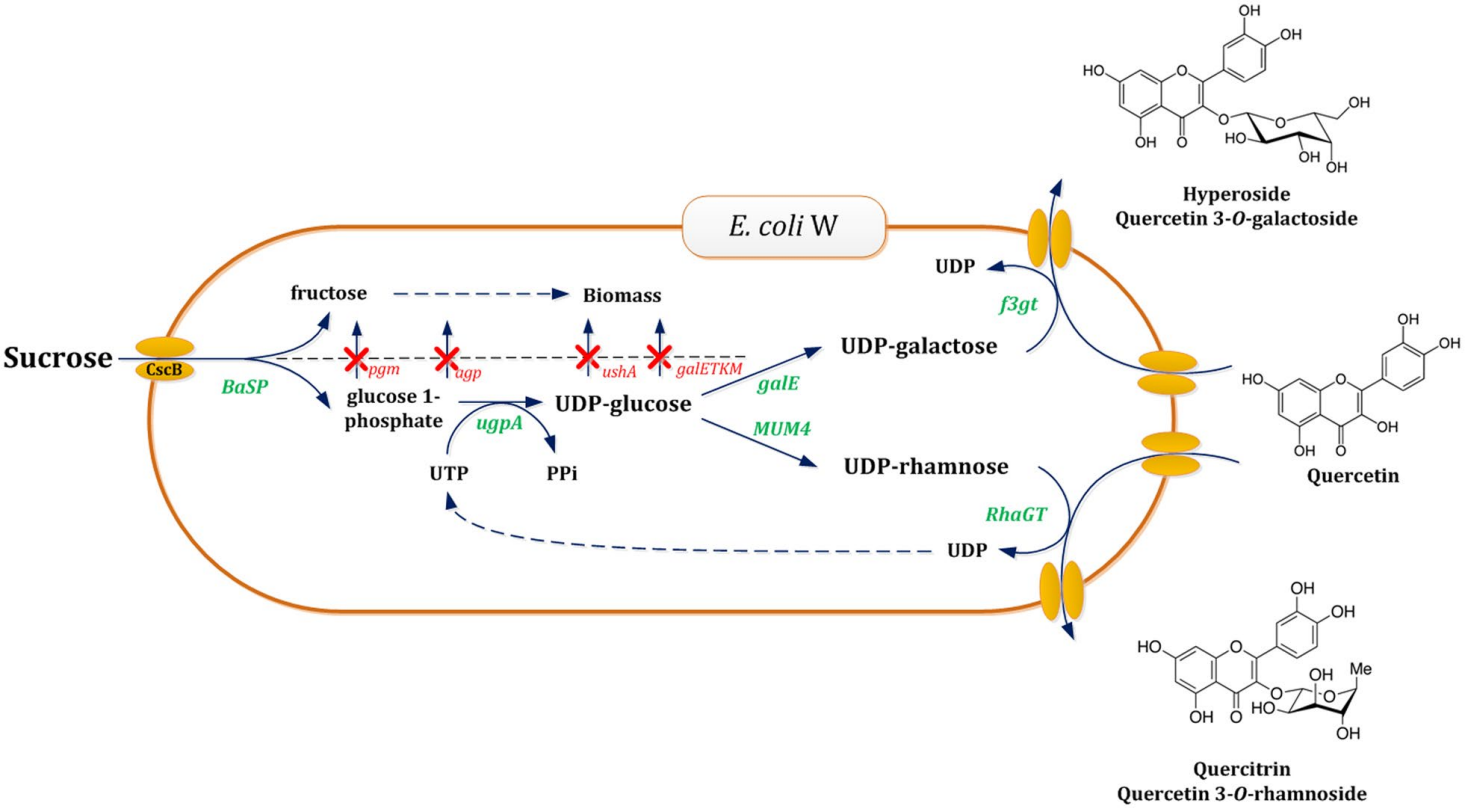
Transformation of *E. coli* W into a sucrose-based galactosylation and rhamnosylation platform. The metabolic engineering strategy applied makes use of several gene deletions (indicated in *red*) and overexpressions of genes (indicated in *green*). The rational of a split metabolism is applied, whereby sucrose is divided by sucrose phosphorylase (BaSP) in fructose to be used for growth and a glucose 1-phosphate as activated precursor for UDP-glucose. The latter is a universal pivot molecule for the formation of UDP-galactose and UDP-rhamnose, interconversions catalyzed by the enzymes GalE and MUM4, respectively. To ensure growth-coupled production, various genes, involved in the metabolization of these UDP-sugars and their precursors, were knocked out (shown in *red*). The production of the bioactive quercetin glycosides hyperoside and quercitrin was chosen to evaluate the versatility of the engineered production platform. Finally, the introduction of either the glycosyltransferase F3GT or RhaGT ensures efficient galactosylation or rhamnosylation, respectively

In recent years flavonoids have garnered much attention from various application domains because of the various beneficial effects on human health that have been attributed to them, such as anticancer [3] and antioxidant [4] to anti-inflammatory [5], antimicrobial [6] and antiviral [6, 7] effects. As final step in their biosynthesis, flavonoids are often glycosylated which has a profound effect on their solubility, stability and bio-activity [8, 9]. For example, the best studied flavonol quercetin, which makes up to 75 % of our daily flavonoid intake, predominantly occurs as different glycosides. Over 350 different quercetin glycoforms have been reported to date with varying pharmacological properties [10, 11]. In this context, hyperoside (quercetin 3-*O*-galactoside) and quercitrin (quercetin 3-*O*-rhamnoside) (Fig. 1) have gained a lot of attention as valuable products for the pharmaceutical industry e.g., as powerful antioxidants with cytoprotective effects [12–15] and as promising antiviral agents that block replication of the influenza virus [16] or inhibit the viruses hepatitis B [17] and SARS [18]. Furthermore, they have been attributed with anti-inflammatory [19, 20], antidepressant [21, 22], apoptotic [23] and antifungal [24] activities, rendering them interesting therapeutics resulting in a steadily increasing market demand.

To date, the majority of quercetin and its glycosides are extracted from plant material, which is generally a laborious and low-yielding process requiring many purification steps [25]. In vitro plant cell cultures or engineered plants can be used to overcome the low yields and improve production [26–28], however since metabolic engineering of plants is both very controversial and still in its infancy [29], this approach is often restricted to small-scale production. Although chemical synthesis of quercetin (glycosides) has proven to be feasible [30–32], stereoselective formation of glycosidic linkages is often hampered by the presence of various reactive groups [33], which requires many protecting and deprotecting steps [34]. In addition, the generation of toxic waste and a low atom-efficiency [35] render these production processes neither sustainable nor economically viable.

As a result, in the last two decades enormous efforts have been invested in the development of alternative production methods for these specialized (secondary) plant metabolites [36]. Advances in the fields of protein engineering, systems and synthetic biology have accelerated these efforts to transform model organisms like *Escherichia coli* and *Saccharomyces cerevisiae* in real microbial cell factories for the sustainable production of flavonoids [37–39]. Subsequently, strategies for the in vivo glycosylation of flavonoids have also been developed. These are typically based on both the overexpression of specific glycosyltransferases, which transfer a sugar residue from an activated nucleotide sugar to an aglycon in a stereo- and regioselective way, and the engineering or introduction of the targeted nucleotide sugar pathway. In this way, various quercetin glycosides have already been produced in *E. coli* such as the naturally occurring 3-*O*-glucoside [40], 3-*O*-xyloside [41] and 3,7-*O*-bisrhamnoside [42], or the new-to-nature quercetin 3-*O*-(6-deoxytalose) [43]. However, despite these engineering efforts, the reported product rates and titers are still in the milligram range, rendering these microbial production hosts unsuitable for industrial applications. The developed production processes are typically biphasic bioconversion processes using resting cells, which makes it difficult to improve production rates [44]. Furthermore, such systems often entail expensive growth media or the addition of enzyme inducers, making the overall process very costly.

To tackle these problems, we previously developed an efficient platform for the glucosylation of small molecules in *E. coli* W [45]. Through metabolic engineering, a mutant was created which couples the production of glucosides to growth, using sucrose as a cheap and sustainable carbon source. By introducing the sucrose phosphorylase from *Bifidobacterium adolescentis* (BaSP) sucrose can be split into fructose to be used for growth purposes and glucose 1-phosphate (glc1P) to be used as precursor for UDP-glucose (UDP-glc) formation (Fig. 1). To impede the conversion of glc1P into biomass precursors, several endogenous genes involved in its metabolization such as phosphoglucomutase (*pgm*) and glucose-1-phosphatase (*agp*) were knocked out. Subsequently, glc1P can efficiently be channeled towards UDP-glc by overexpressing the uridylyltransferase from *Bifidobacterium bifidum* (*ugpA*). Metabolization of UDP-glc is prevented by knocking out the UDP-sugar hydrolase (*ushA*) and the galactose operon (*galETKM*). However, in view of the pivotal role of UDP-glc in the production of a large variety of UDP-sugars, this glucosylation system can easily be extended towards other UDP-sugars, such as UDP-galactose (UDP-gal), UDP-rhamnose (UDP-rha) and UDP-glucuronate.

In the present contribution, this previously developed *E. coli* W-based glucosylation platform is transformed into a platform for galactosylation and rhamnosylation (Fig. 1), whose potential is demonstrated using the galactosylation and rhamnosylation of exogenously fed quercetin yielding hyperoside and quercitrin, respectively, as case study.

## Results and discussion

### Using *E. coli* W as a host for in vivo glycosylation

*Escherichia coli* W is a fast-growing non-pathogenic strain which tolerates osmotic stress, acidic conditions, and can be cultured to high cell densities, making it an attractive host for industrial fermentations [46]. Moreover, *E. coli* W is able to grow on sucrose as sole carbon source [46], which is an emerging feedstock for the production of bio-products. Hence, *E. coli* W was selected as host for sucrose-based in vivo glycosylation. Prior to the production of the glycosides hyperoside and quercitrin in *E. coli* W, the toxicity of their aglycon quercetin was investigated. To this end, the wild type (WT) strain was grown on minimal sucrose medium containing different concentrations of quercetin (0, 0.15 and 1.5 g/L). The specific growth rates (h^−1^) (0.96 ± 0.06, 0.92 ± 0.05 and 0.87 ± 0.06, respectively) were not significantly different (p_ANOVA_ = 0.12) nor from the one previously determined for the WT [45] (*p* = 0.69, *p* = 0.98 and *p* = 0.68, respectively). On the other hand, the optical density at 600 nm after 24 h incubation (6.36 ± 0.12, 5.18 ± 0.16 and 4.77 ± 0.20, respectively) was lower (about 20 %) when quercetin was added (*p* = 0.0002 and *p* = 0.0001). No significant difference in optical density could be observed between 0.15 and 1.5 g/L quercetin (*p* = 0.14). In view of the above, it was opted to add 1.5 g/L quercetin to evaluate the potential of the developed glycosylation platform.

To evaluate the in vivo glycosylation potential, strains sGAL1 and sRHA1, which constitutively express the flavonol 3-*O*-galactosyltransferase from *Petunia hybrida* and the flavonol 3-*O*-rhamnosyltransferase from *A. thaliana*, respectively, were cultured in minimal medium with 1.5 g/L of quercetin for 16 h. TLC analysis of the supernatants of both cultures yielded two new yellow product spots. The TLC spot obtained from the sGAL1 culture, which had the same retention time as the hyperoside standard (R_f_ = 0.5), was subsequently purified and analyzed. Both NMR and MS analysis confirmed the production of quercetin 3-*O*-galactoside. However, the product spot obtained from the sRHA1 culture had a different retention factor (R_f_ = 0.55) than the quercitrin standard (R_f_ = 0.74), and was identified as isoquercitrin (quercetin 3-*O*-glucoside). As opposed to other reports on wild type *E. coli* strains expressing RhaGT, which simultaneously produced quercitrin (quercetin 3-*O*-rhamnoside) and isoquercitrin [47, 48], no rhamnoside could be detected. Examination of the *E. coli* W genome revealed that the gene cluster responsible for the endogenous production of dTDP-rhamnose, which functions as an alternative rhamnosyldonor for RhaGT in *E. coli* B and K12 derivatives [47], was not present [46, 49].

**Fig. 2:**
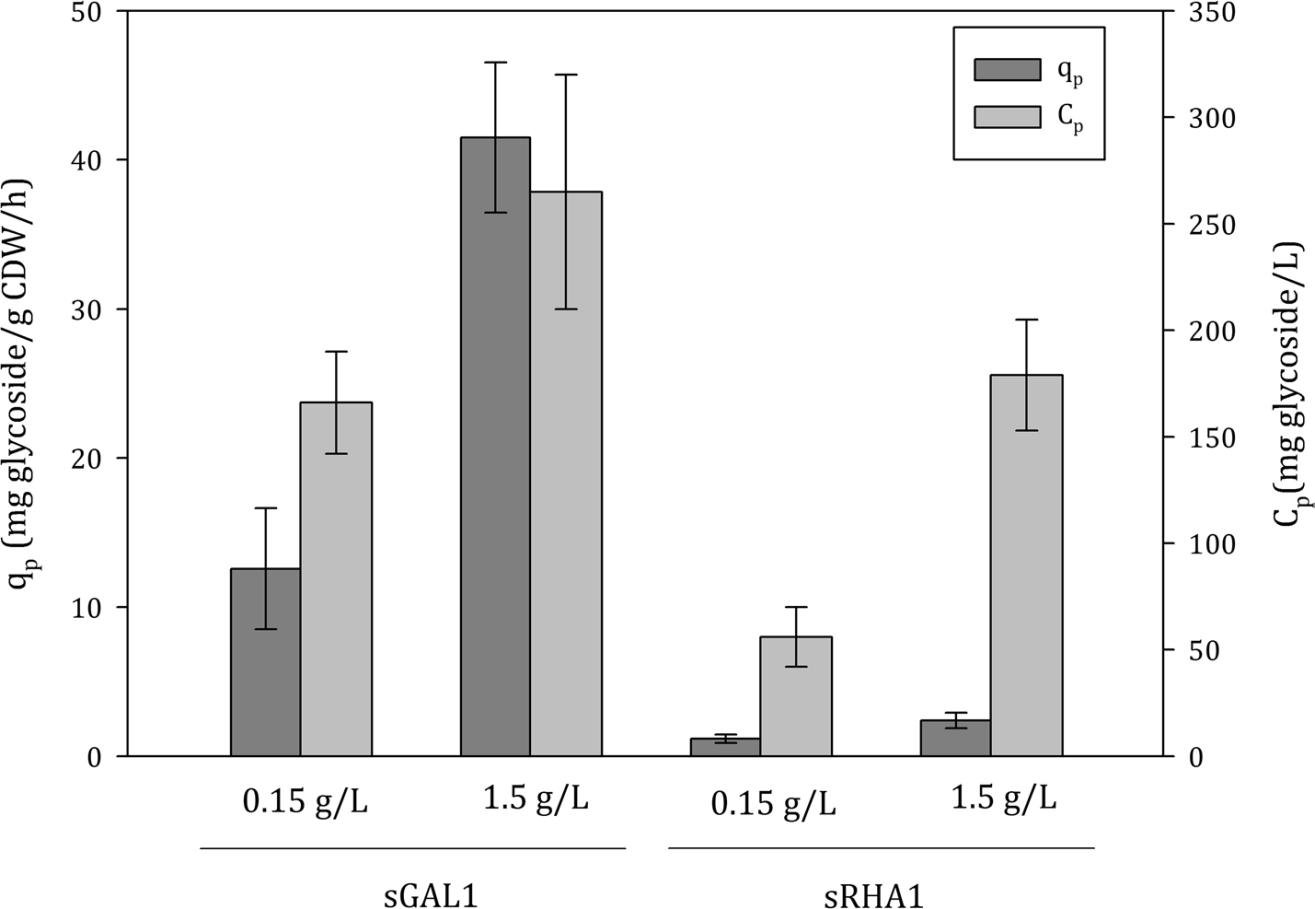
Comparison of the specific glycoside productivities (q_p_) and glycoside titers (C_p_) for strains sGAL1, which produces the 3-*O*-galactoside, and sRHA1, which produces the 3-*O*-glucoside, when grown for 30 h on minimal medium containing 0.15 or 1.5 g/L of quercetin. *Error bars* represent standard deviations

In a follow-up experiment, sGAL1 and sRHA1 were grown on minimal medium with two different concentrations (0.15 and 1.5 g/L) of quercetin. Growth and glycoside formation were monitored during 30 h. The final titers (C_p_) and specific productivities (q_p_) are shown in Fig. 2. Remarkably, an increase in quercetin concentration resulted in a two to threefold increase in productivity and titer, indicating that quercetin supply is rate-limiting and crucial for efficient in vivo glycosylation. However, while sGAL1 continuously produced hyperoside during the exponential phase, which is also reflected in the relatively high specific productivity, sRHA1 only started to accumulate significant amounts of isoquercitrin at the end of the exponential phase. This production start coincides with a reduction in specific growth rate, which dropped from 0.35 ± 0.04 to 0.06 ± 0.01 h^−1^.

### Construction of an advanced sucrose-based glycosylation strain

**Fig. 3:**
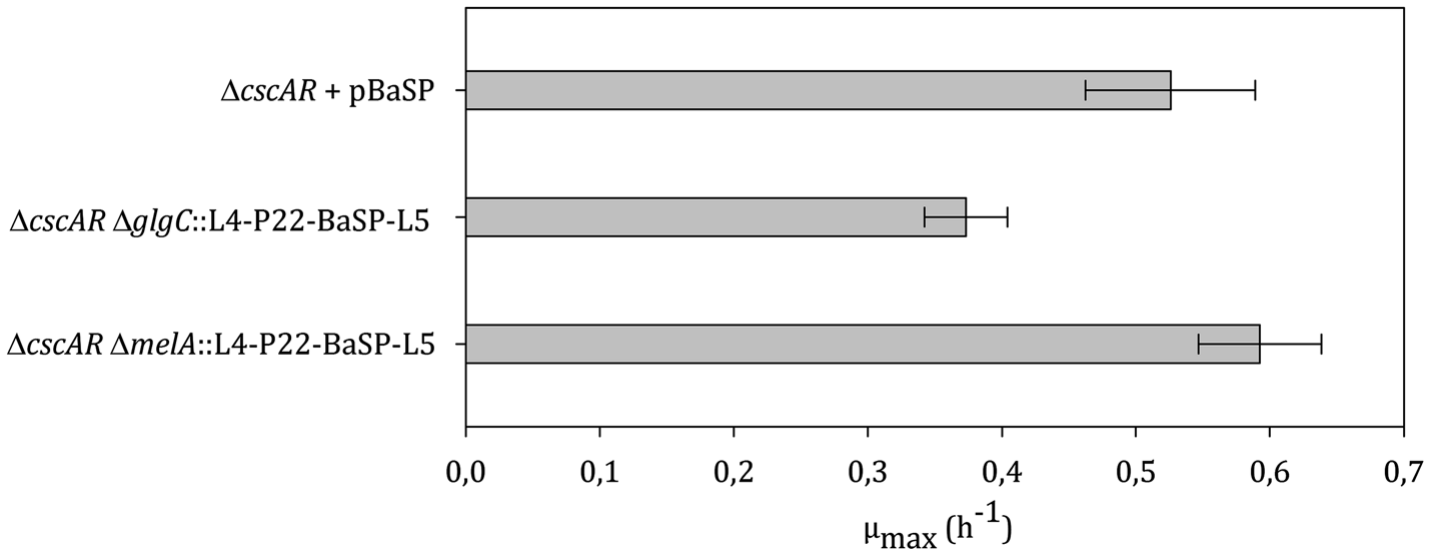
Effect of the chromosomal integration locus of the knockin of *BaSP* on the growth rate. Strains were grown in shake flasks and the resulting maximal growth rates (µ_max_) were compared with *E. coli* W Δ*cscAR* with plasmid-based *BaSP* expression (+pBaSP). *Error bars* represent standard deviations

As described in detail in the Background section, we previously metabolically engineered *E. coli* W to create a platform for in vivo glucosylation of small molecules [45]. In the original base glucosylation strain, sucrose phosphorylase encoded by *BaSP* was located on a medium-copy plasmid and transcribed from a medium-strong constitutive promoter (P22) [50]. For reasons of comparison and flexibility, it was opted to integrate *BaSP* in the genome of *E. coli* W. In addition, chromosomal integration is advantageous because of a significant increase in gene stability. Since the level of gene expression can considerably be impacted by the genome integration site [51] due to structural differences such as supercoiling DNA regions, two different DNA sites were assessed for *BaSP* integration, i.e., *melA* and *glgC*, which encode an α-galactosidase and a glucose-1-phosphate adenylyltransferase, respectively. To this end, an adapted knockin-knockout procedure for large DNA fragments was applied, which is schematically shown in Additional file 1: Figure S2. *BaSP* under control of promoter P22 was knocked in at the two different loci in *E. coli* W Δ*cscAR*, which resulted in the *E. coli* W strains Δ*cscAR* Δ*melA*::L4-P22-*BaSP*-L5 and Δ*cscAR* Δ*glgC*::L4-P22-*BaSP*-L5. Their maximal specific growth rate (µ_max_) on minimal sucrose medium, which is shown in Fig. 3, was compared to the original strain Δ*cscAR* + pBaSP. The influence of the knockin locus on the maximal specific growth rate is clear. Interestingly, integration at the *melA* locus resulted in a strain with a µ_max_ which was not significantly different from the reference strain Δ*cscAR* + pBaSP. In view of the latter and considering the aimed growth-coupled production, it was opted to integrate *BaSP* at the *melA* locus leading to the final production base strain *E. coli* W Δ*cscAR* Δ*pgm* Δ*agp* Δ*ushA* Δ*lacZYA*::P22-*lacY* Δ*galETKM* Δ*melA::*L4-P22-*BaSP*-L5 (sGLYC) as shown in Table 1.

**Table 1:** Plasmids and strains used in this study

Strain or plasmid	Description	References
*Plasmids*		
pBaSP	pUC57 vector expressing *BaSP* from *B. adolescentis* under control of P22	De Bruyn et al. [45]
pGalE	pUC57 vector expressing *galE* from *E. coli* under control of P22	This study
pGalE2	pUC57 vector expressing *galE2* from *B. bifidum* under control of P22	This study
pMUM4	pUC57 vector expressing codon optimized *MUM4* from *A. thaliana* under control of P22	GeneArt^®^
pF3GT	pUC57-Kan vector expressing codon optimized *f3gt* from *Petunia hybrida* under control of P22	GeneArt^®^
pRhaGT	pUC57-Kan vector expressing codon optimized *RhaGT* from *A. thaliana* under control of P22	GeneArt^®^
pBaSP/VvGT2/UgpA	pCX-Kan vector expressing *BaSP,* *vvGT2* and *ugpA* under control of P22	De Bruyn et al. [45]
pGalE/F3GT/UgpA	pCX-Kan vector expressing *galE,* *f3gt* and *ugpA* under control of P22	This study
pGalE2/F3GT/UgpA	pCX-Kan vector expressing *galE2,* *f3gt* and *ugpA* under control of P22	This study
pMUM4/RhaGT/UgpA	pCX-Kan vector expressing *MUM4,* *RhaGT* and *ugpA* under control of P22	This study
pKD46	λ Red recombinase expression, Amp^r^	Datsenko and Wanner [62]
pCP20	FLP recombinase expression, Amp^r^, Cm^r^	Datsenko and Wanner [62]
pKD3	Cm cassette template, Cm^r^, Amp^r^	Datsenko and Wanner [62]
pKD4	Kan cassette template, Kan^r^, Amp^r^	Datsenko and Wanner [62]
*Strains*		
*E. coli* DH5α	General cloning host	Coli Genetic Stock Center
*E. coli* W	*Escherichia coli* W ATCC 9637	BCCM/LMG
*E. coli* W ∆*cscAR*	*E. coli* W with *cscAR*-deleted	De Bruyn et al. [45]
*E. coli* W ∆*cscAR* ∆*melA*::L4-P22-*BaSP*-L5	*E. coli* W with *cscAR* and *melA* deleted and L4-P22-BaSP-L5 integrated in the genome	This study
*E. coli* W ∆*cscAR* ∆*glgC*::L4-P22-*BaSP*-L5	*E. coli* W with *cscAR* and *glgC* deleted and L4-P22-BaSP-L5 integrated in the genome	This study
*E. coli* W ∆*cscAR* ∆*pgm* ∆*agp* ∆*ushA* Δ*lacZYA*::P22-*lacY* Δ*galETKM*	*E. coli* W with *cscAR*, *pgm*, *agp*, *ushA,* *lacZYA,* *melA* and *galETKM* deleted and P22-*lacY* integrated in the genome	De Bruyn et al. [45]
*E. coli* W ∆*cscAR* ∆*pgm* ∆*agp* ∆*ushA* Δ*lacZYA*::P22-*lacY* Δ*galETKM* Δ*melA*::P22-BaSP (sGLYC)	*E. coli* W with *cscAR*, *pgm*, *agp*, *ushA,* *lacZYA,* *melA* and *galETKM* deleted and P22-*lacY* and L4-P22-BaSP-L5 integrated in the genome	This study
sGAL1	*E. coli* W + pF3GT	This study
sGAL2	sGLYC + pGalE/F3GT/UgpA	This study
sGAL3	sGLYC + pGalE2/F3GT/UgpA	This study
sRHA1	*E. coli* W + pRhaGT	This study
sRHA2	sGLYC + pMUM4 + pRhaGT	This study
sRHA3	sGLYC + pMUM4/RhaGT/UgpA	This study

### Enhanced production of bio-active quercetin glycoforms

In nature, UDP-glc serves as a pivot molecule in the formation of a variety of UDP-sugars [44]. For example, using the interconverting enzymes UDP-glucose 4-epimerase (GalE) and UDP-rhamnose synthase (MUM4) UDP-glc can be converted to UDP-gal and UDP-rha, respectively. Though GalE is natively present in *E. coli* W an alternative homologous epimerase (GalE2) from *B. bifidum* was also selected and cloned due to the tight and complex regulation of GalE expression in *E. coli* W. On the other hand, UDP-rhamnose synthesis is restricted to plants. Due to lack of the *rfb* cluster [46] *E. coli* W is even unable to form endogenous dTDP-rhamnose as alternative rhamnosyl donor. Hence, the *MUM4* gene from *A. thaliana* was expressed from plasmid pMUM4 to achieve UDP-rhamnose formation in *E. coli* (Fig. 1).

**Fig. 4:**
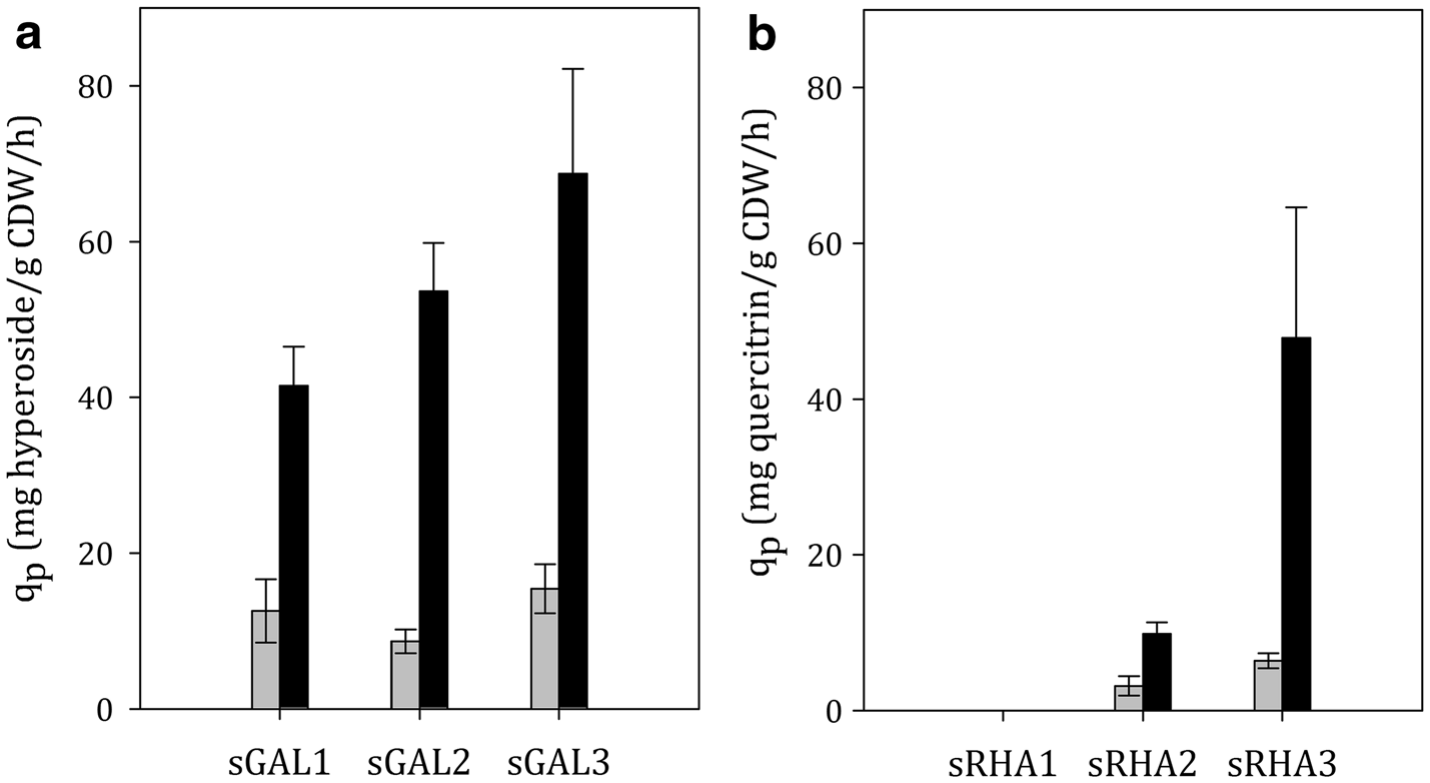
Comparison of the specific productivity q_p_ of the sGAL (**a** hyperoside formation) and sRHA (**b** quercitrin formation) strains. Strains were grown on minimal medium containing 0.15 g/L (*gray bars*) and 1.5 g/L (*black bars*) quercetin. *Error bars* represent standard deviations

The constructed galactosylation (sGAL) and rhamnosylation (sRHA) strains were grown on minimal medium with two levels (0.15 and 1.5 g/L) of quercetin. Growth and production were monitored to determine the specific productivities, as shown in Fig. 4. Again, higher extracellular quercetin concentrations resulted in a fivefold increase in q_p_. However, no significant difference in productivity was observed between sGAL2 and sGAL3 at 1.5 g/L quercetin, indicating that UDP-galactose formation is as efficient with both GalE homologs and not likely the rate limiting step. With sGAL3, the highest hyperoside productivity (68.7 mg/g CDW/h) and titer (0.94 g/L) were obtained, the latter being 3.5-fold higher compared to sGAL1.

In contrast to sRHA1, TLC analysis of the supernatant of the cultures of sRHA2 and sRHA3 resulted in a product spot with a retention factor that corresponds to quercitrin, which was confirmed by MS analysis, thus showing in vivo activity of MUM4. A quercitrin titer of 1.18 g/L and specific productivity of 47.8 mg/g CDW/h were obtained after 30 h incubation of sRHA3 when 1.5 g/L quercetin was added to the medium, which corresponded to a 53 % conversion. Also 51 mg/L of isoquercitrin was produced extracellularly which corresponds with a quercitrin:isoquercitrin production ratio of 24:1. This suggests the preference of RhaGT for UDP-rhamnose when different UDP-sugar donors are present. Possible explanations for the significantly lower specific productivity (fivefold decrease) of sRHA2 as compared to sRHA3 are either a higher metabolic burden [52] caused by the two plasmid system or a too limited activity of the native GalU, which could be insufficient for adequate UDP-glc formation [45].

**Fig. 5:**
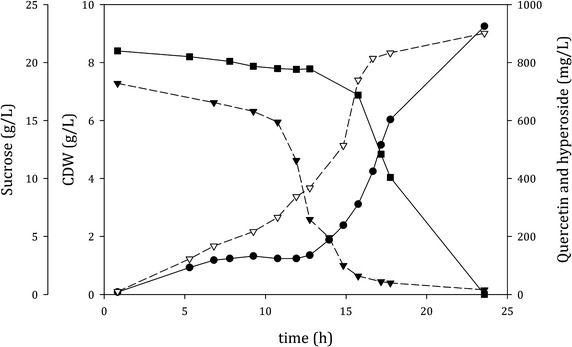
Production of hyperoside (*open inverted triangle*) in a 1-L bioreactor on minimal medium containing 0.76 g/L quercetin (*filled inverted triangle*) by strain sGAL3. Cell dry weight (*filled circle*) was measured together with extracellular sucrose (*filled square*)

To demonstrate the scalability of the developed bio-process, strain sGAL3 was cultured in a 1-L bioreactor, which also ensures a constant pH set at 6.80 and avoid oxygen limitation. A detailed overview of the consumption of sucrose, growth and hyperoside production is given in Fig. 5. After a lag-phase, the strain displayed a growth rate of 0.32 ± 0.02 h^−1^ while simultaneously producing hyperoside. The observed specific productivity (65.9 ± 2.6 mg/g CDW/h) was comparable to the one obtained on shake flask scale. When nearly all quercetin was converted, hyperoside formation slowed down, which can be explained either by the observed correlation between quercetin concentration and q_p_, or by the reported reversibility of F3GT [53]. It is likely that further improvements in titer and productivity can be realized by optimizing the supply of quercetin using a fed-batch system.

**Fig. 6:**
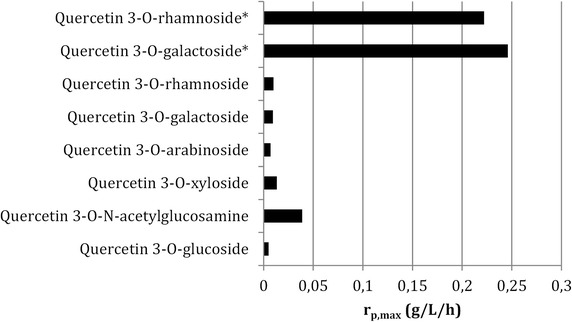
Comparison of the production rates obtained using the developed galactosylation and rhamnosylation platform (*asterisk*) with those reported in the literature for quercetin 3-*O*-rhamnoside [47], quercetin 3-*O*-galactoside [54], quercetin 3-*O*-arabinoside [66], quercetin 3-*O*-xyloside [66], quercetin 3-*O*-*N*-acetylglucosamine [67] and quercetin 3-*O*-glucoside [40]

To the best of our knowledge, the results obtained in this study with the engineered sGAL and sRHA strains for the production of hyperoside and quercitrin are the highest reported to date both in terms of titer and production rate. The maximal production rate obtained in this contribution was 6 to 50-fold higher compared to the maximal production rates (r_p,max_) of processes reported in the literature [47, 54] as is illustrated in Fig. 6.

The increased performance, in terms of titer and productivity, obtained with the developed platform can be attributed to the use of a split metabolism in combination with optimally rerouting the flux from glucose 1-phosphate towards UDP-galactose and UDP-rhamnose. The undesired conversion of the activated sugars into biomass is impeded by gene deletions, which guarantees a high product yield. In addition, since biomass formation, which is fueled by the fructose moiety of sucrose, and glycoside synthesis go hand in hand and subsequently are performed at the same time at a high rate, a high productivity is equally guaranteed (one-step fermentation process).

### Beyond quercetin: in vivo glycosylation of other flavonols

Besides quercetin also other flavonols such as kaempferol, fisetin, morin and myricetin significantly contribute to our daily flavonoid intake, which also have extremely diverse beneficial effects [55, 56]. As the sugar moiety is a major determinant of the intestinal absorption of dietary flavonoids and their subsequent bioactivity [57, 58], the potential of the created *E. coli* W mutants towards both galactosylation and rhamnosylation of various flavonols was investigated.

To this end, strains sGAL3 and sRHA3 were grown in tubes with 5 mL minimal medium, each containing 1.5 g/L of either kaempferol, myricetin, morin or fisetin. Growth and production were monitored over 48 h and various spots were observed on TLC with similar retention factors as hyperoside and quercitrin. Mass spectrometry was used to identify the compounds produced, which confirmed the in vivo galactosylation of myricetin, kaempferol, morin and fisetin (Table 2). All compounds occurred with an m/z of [M + 114], due to complexation with trifluoroacetic acid from the mobile phase. The galactoside of morin was produced at a slow rate, which is in accordance to the very low in vitro activity of F3GT towards this flavonol [53]. A possible explanation for this limited activity may be the presence of an unusual hydroxyl group at the 2′ position, which may sterically hinder deprotonation and consequent galactosylation of morin at hydroxyl group 3 [59].

**Table 2:** Galactosylation and rhamnosylation potential of strains sGAL3 and sRHA3 respectively towards other flavonols

Flavonol	3-*O*-galactoside		3-*O*-rhamnoside	
	C_p_ (mg/L)	q_p_ (mg/gCDW/h)	C_p_ (mg/L)	q_p_ (mg/gCDW/h)
Kaempferol	84 ± 14	3.46 ± 0.86	416 ± 37^a^	12.1 ± 1.4
Myricetin	52 ± 7.1	2.88 ± 0.22	72.3 ± 9.1	2.8 ± 0.5
Morin	34 ± 5.8	1.65 ± 0.15	116 ± 21^b^	2.5 ± 1.5
Fisetin	134 ± 22	9.32 ± 0.55	403 ± 31^c^	11.3 ± 0.9

Specific productivity (q_p_) and titer (C_p_) reached after 48 h of incubation are shown

*ND* not detected

^a^3-*O*-glucoside was also detected at 52 ± 17 mg/L

^b^3-*O*-glucoside was also detected at 21.7 ± 6.2 mg/L

^c^3-*O*-glucoside concentration was lower than 5 mg/L

Incubation of sRHA3 with the different flavonols investigated showed two distinct glycoside spots on TLC, which corresponded to the 3-*O*-rhamnoside and 3-*O*-glucoside. Kaempferol proved to be the best substrate for RhaGT and was predominantly rhamnosylated (8:1 ratio), with a titer exceeding 400 mg/L, which is twofold higher than previously reported [47]. Fisetin on the other hand was efficiently glucosylated, yet the formation of its rhamnoside was not as efficient, with a titer below 5 mg/L. A similar preference towards glucoside formation was also observed with myricetin and morin, which indicates that the positioning of the hydroxyl groups is the determining factor for glycosylation with RhaGT. The production of the desired rhamnosides, galactosides or glucosides may be improved considerably by using UGTs that are more specific towards certain flavonols and UDP-sugars. Transformation of the corresponding UGTs in the developed in vivo glycosylation strains presents a promising alternative for the large-scale production of various flavonol glycoforms, which are to date mainly extracted from plant material.

On the other hand, due to the pivotal role of UDP-glc, various other UDP-sugars can be formed in vivo (e.g. UDP-glucuronate, UDP-xylose, UDP-arabinose). In combination with the modularity of the developed glycosylation platform, which permits rapid introduction of any UGT or UDP-sugar pathway, virtually any glycoside can be produced. Hence, this demonstrates that the proposed microbial platform is a robust, versatile and efficient microbial cell factory for the glycosylation (e.g. glucosylation, rhamnosylation, galactosylation) of small molecules. Although obtained productivities are the highest reported today and compete with the current production processes, further improvement can be limited due to solubility issues of the aglycon or of the glycoside. To this end follow-up research can focus on further metabolic engineering (e.g. introduction of the aglycon pathway allowing in vivo gradually production of the aglycon) or on process optimization [e.g. 2-phase (bilayer) fermentation which enables in situ recovery] to improve these issues.

## Conclusions

In this contribution, a biotechnological platform was developed for the galactosylation and rhamnosylation of small molecules, such as secondary metabolite natural products, starting from a previously created glucosylation host. To this end, the routes to convert UDP-glucose into UDP-galactose and UDP-rhamnose were introduced by expressing a UDP-glucose epimerase (*galE*) and a UDP-rhamnose synthase (*MUM4*), respectively. As a proof of concept, the bio-active flavonol quercetin was selected for galactosylation and rhamnosylation, yielding hyperoside (quercetin 3-*O*-galactoside) and quercitrin (quercetin 3-*O*-rhamnoside), respectively. Next, the flavonol 3-*O*-galactosyltransferase (F3GT) from *Petunia hybrida* and the flavonol 3-*O*-rhamnosyltransferase from *Arabidopsis thaliana* (RhaGT) were overexpressed in the metabolically engineered *E. coli* W mutants. The strains created were able to produce 940 mg/L of hyperoside and 1176 mg/L of quercitrin at specific production rates of 68.7 mg/g CDW/h and 47.8 mg/g CDW/h, respectively, which are the highest reported to date. Interestingly, both GTs showed in vivo activity towards other dietary flavonols, whereby for example over 400 mg/L of kaempferol 3-*O*-rhamnoside could be formed extracellularly.

## Methods

### Materials and molecular agents

All plasmids used were constructed using Gibson assembly [60] or CLIVA [61]. All PCR fragments were amplified using Q5 polymerase from New England Biolabs (Ipswich, Massachusetts). Oligonucleotides were purchased from IDT (Leuven, Belgium). The plasmids and bacterial strains used in this study are listed in Table 1. A list of primers for the creation of gene knockouts/knockins and for the cloning of the expression plasmids is given in Additional file 2: Table S1. *E. coli* DH5α was used for plasmid cloning and propagation, while *E. coli* W was used for expression of the production plasmids and the creation of gene knockouts and knockins. Hyperoside, quercitrin, isoquercitrin, kaempferol and myricetin were purchased from Carbosynth (Berkshire, UK). All other chemicals used were purchased from Sigma Aldrich (Germany) unless otherwise indicated.

### Creation of the production plasmids

The expression plasmids for the prod uction of hyperoside and quercitrin were constructed as depicted in Additional file 3: Figure S1A−C. Plasmids pF3GT, pRhaGT and pMUM4 were synthesized by GeneArt^®^ (Life Technologies). The *f3gt* sequence [Genbank: AF165148] from *Petunia hybrida*, and *MUM4* [Genbank: AT1G53500] and *RhaGT* [Genbank: AF360160] from *A. thaliana* were codon optimized for *E. coli* (gene sequences are given in Figure S1D). The *galE* [Genbank: JW0742] and *galE2* [Genbank: KJ543703] sequences were amplified from the genomic DNA of *E. coli* and *Bifidobacteriumbifidum*, respectively. CLIVA assembly resulted in the intermediary plasmid pBaSP/F3GT/UgpA (Figure S1A), which was subsequently used for the amplification of the F3GT/UgpA backbone. Gibson assembly of the GalE or GalE2 inserts with this backbone resulted in the final galactosylation plasmids pGalE/F3GT/UgpA and pGalE2/F3GT/UgpA, respectively (Figure S1B). Similarly, MUM4 and RhaGT were introduced using a 3-pieces Gibson assembly (Figure S1C), resulting in the final rhamnosylation plasmid pMUM4/RhaGT/UgpA.

### Creation of the *E. coli* W production mutants

The overall *E. coli* W knockout mutants were created using the one step deletion system of Datsenko and Wanner [62]. The strategy for chromosomal integration of *BaSP* under control of the constitutive promoter P22 flanked by L4 and L5 at the *melA* and *glgC* loci is depicted and explained in Additional file 1: Figure S2. Transformants were plated on minimal sucrose medium agar plates and grown overnight for screening. The in-house strain *E. coli* W ∆*cscAR* ∆*pgm* ∆*agp* ∆*ushA* Δ*lacZYA*::P22-*lacY* Δ*galETKM* [45] was used for the chromosomal integration of L4-P22-*BaSP*-L5 at the *melA* site, yielding the base strain sGLYC (Table 1). This strain and the *E. coli* W wild type were transformed with the production plasmids described above, resulting in the galactosylation (sGAL) and rhamnosylation (sRHA) strains given in Table 1.

### Media

Composition of LB and minimal sucrose medium was described previously [45]. Minimal medium agar plates with sucrose (50 g/L) had the same composition as minimal sucrose medium, but contained additionally 15 g/L of agarose. The agarose and salts were autoclaved separately at 121 °C for 21 min. Sucrose was filter sterilized through a 0.22 µm corning filter (Fisher, Belgium) and heated for 1 min in a microwave oven at 800 W prior to mixing it with the warm agarose and salt solutions. 1 mL/L of mineral solution [45] was sterilely added prior to pouring the plates.

### Growth in shake flasks and sampling

*Escherichia coli* W mutant precultures were grown in 5 mL LB medium with the antibiotics (50 μg/mL kanamycin or carbenicillin) required for maintenance and selection of the plasmids. The cultures were grown for 16 h at 37 °C and 200 rpm and used for the 2 % inoculation of 100 mL minimal sucrose medium in 500 mL shake flasks. For the production of hyperoside and quercitrin, quercetin was added to the minimal medium at a concentration of 0.15 or 1.5 g/L. Growth conditions were the same as previously described [45]. Samples were taken at regular intervals from the broth and, after centrifugation, the supernatant was used for the analysis and quantification of sugars. For the analysis of quercetin and its glycosides, 200 µL of the culture was collected and extracted with 800 µL ethyl acetate. The organic layer was collected, evaporated in a SpeedVac™ vacuum concentrator (Thermo Fisher, USA) and dissolved in 200 µL of DMSO for HPLC quantification.

### Growth in bioreactors

The bioreactor set-up and fermentation conditions used are the same as previously described [45]. Production experiments were performed on minimal sucrose medium without MOPS buffer and with the addition of quercetin as acceptor.

### Product analysis and quantification

Culture samples were primarily analyzed by TLC on Silica gel 60 F_254_ precoated plates (Merck, Germany). All plates were run in a closed TLC chamber and developed using standard visualization techniques and agents: UV fluorescence (254 nm) or by staining with 10 % (v/v) H_2_SO_4_ and subsequent charring. The mobile phase for detecting the various flavonols and corresponding glycosides consisted of an ethyl acetate:acetic acid:formic acid:water (100:11:11:27 v/v) mixture [63]. Product spot intensities of other flavonol glycosides were processed and quantified using ImageJ [64]. HPLC quantification of sucrose, fructose and glucose was performed using an X-bridge Amide column (35 μm, Waters, USA) as described previously [45]. Quercetin, hyperoside, quercitrin and isoquercitrin were detected with the method described by Pandey et al. [41] using a Varian HPLC system (Agilent technologies, California). Mass spectrometry for determination of the various flavonol glycosides was performed with a Micromass Quattro LC (McKinley Scientific, USA). Detection was performed in negative mode ESI-224 MS with a capillary voltage of 2.53 kV, a cone voltage of 20 V, cone and desolvation gas flows of 93 and 420 L/h, and source and cone temperatures of 150 and 350 °C, respectively.

### Purification and structural elucidation of compounds

Quercetin glycosides were extracted from the broth with an equal volume of ethyl acetate after which the organic layer was evaporated to dryness. The remaining product was dissolved in the solvent system described above and run on a preparative TLC plate. The band containing hyperoside (R_f_ 0.53) or quercitrin (R_f_ 0.75) was scraped off, extracted with ethyl acetate and evaporated to yield a bright yellow powder. Products were confirmed by NMR. Spectra were reported elsewhere [47, 65].

## Additional files

**Additional file 1: Figure S2.** Cloning strategy for KO KI BaSP
**Additional file 2: Table S1.** Primers for the construction of knockouts and plasmids
**Additional file 3: Figure S1.** Cloning strategy for plasmids of the galactosylation and rhamnosylation platform
