# Supplementary material for: Metabolic engineering of Escherichia coli into a versatile glycosylation platform: production of bio-active quercetin glycosides
Source: Microb Cell Fact. 2015 Sep 16;14:138. doi: 10.1186/s12934-015-0326-1 (PMC4573293; doi:10.1186/s12934-015-0326-1)
Supplement: Supplementary file 1 — Additional file 1: Figure S2. Cloning strategy for KO KI BaSP [file 12934_2015_326_MOESM1_ESM.docx]

**Figure S2:** **Cloning strategy for KO/KI of Δ*melA*::*BaSP* and Δ*glgC*::*BaSP***

*BaSP* knockin strategy at two different loci, resulting in two new strains. First, *BaSP* and its surrounding linker sequences, were amplified from the plasmid pUC-L4-P22-BaSP-L5 using PCR. Adjacent DNA regions of the integration spots, genes *melA* and *glgC*, were also amplified from the genome. The primers were designed with the reverse primer of the left border and the forward primer of the right border having an overhanging sequence homologous to respectively linker L4 and L5. Also the forward primer of the left border has an overhang sequence homologous to the right end of the right border. For both the *melA* and *glgC* gene replacement, these 3 pieces were put together using Gibson Assembly. Subsequently, these circular DNA molecules were used as a template to pick up the knockin-knockout constructs containing prolonged homologous regions for recombination.
