# Supplementary material for: Metabolic engineering of Escherichia coli into a versatile glycosylation platform: production of bio-active quercetin glycosides
Source: Microb Cell Fact. 2015 Sep 16;14:138. doi: 10.1186/s12934-015-0326-1 (PMC4573293; doi:10.1186/s12934-015-0326-1)
Supplement: Supplementary file 2 — Additional file 2: Table S1. Primers for the construction of knockouts and plasmids [file 12934_2015_326_MOESM2_ESM.docx]

**Table S1A: Primers for knockouts/knockins**

| **Primers ^a^** | **Oligonucleotide sequences (5’-3’)** |
| --- | --- |
| Fw_L4_start_BaSP_KI | GGCAGGAGTATCGTCCGTAG |
| Rv_L5_tail_BaSP_KI | CTGAAGGCGCTCCTCATAC |
| Fw_glgC::BaSP_L | GACCATGTTCGTCACCGGCCAGTAACATCGCCTTGATAGGCCAGGTTGTG |
| Rv_glgC::BaSP_L | ACAGTTGAAACTACGGACGATACTCCTGCCGAAATGCTACGGAAGTTAGG |
| Fw_glgC::BaSP_R | GTATTCAGCCAGTATGAGGAGCGCCTTCAGGACTAACTCCTTTTTTATCATCTCTGGAAC |
| Rv_glgC::BaSP_R | CGATGTTACTGGCCGGTGAC |
| Fw_melA::BaSP_L | GGATATTCCCTTCTGGTCGCTGGTTCCAAGCGTGATATGACCCTGATTG |
| Rv_melA::BaSP_L | ACAGTTGAAACTACGGACGATACTCCTGCCCTCCTGGCTTGCTTGAATAAC |
| Fw_melA::BaSP_R | GTATTCAGCCAGTATGAGGAGCGCCTTCAGAACGCGACTAAACGCTACTG |
| Rv_melA::BaSP_R | TTGGAACCAGCGACCAGAAG |
| Fw_glgC_KO_BaSP_KI | AAGCCCGCTGGCCATTTCTG |
| Rv_glgC_KO_BaSP_KI | CGCCGCGTTAATCCATCTGC |
| Fw_melA_KO_BaSP_KI | TGCTTCACGCAGGATCTGAG |
| Rv_melA_KO_BaSP_KI | ACAGACAGCCCAACGACATC |

**^a^ Notations KO: knockout, KI: knockin**

**Table S1B: Primers for plasmid constructions**

| **Primers** | **Oligonucleotide sequences (5’-3’)** |
| --- | --- |
| Fw_L4_backbone | ATTTATAAATGAAGCGGCCGCCCGGGATAGACTTCAGGCAGACCACGCTTGAC |
| Rv_L5_Backbone | TAACCATGGGCTAGCATTGC |
| Fw_L5_backbone | ATTTATAAATGAAGCGGCCGCCCGGGATCTGAAGGCGCTCCTCATACTG |
| Rv_L6_Backbone | TAACCATGGGCTAGCGGCCAGCAAAG |
| Fw_L6_backbone | ATTTATAAATGAAGCGGCCGCCCGGGATGTTCCGATGGCGTGCATCAG |
| Rv_L7_Backbone | TAACCATGGGCTAGCAATGC |
| Univ_Fw_primer_P22_insert | ATCCCGGGCGGCCGCTTCATTTATAAATTTC |
| Rv_insert_BaSP | AGTCGGAACGGCAATGCTAGCCCATGGTTATCAGGCGACGACAGGCGGATTG |
| Rv_insert_VvGT2 | GTCCCTTTGCTGGCCGCTAGCCCATGGTTATTAAATTTTCTTTGACTTGCAAACCAGCTCCATAC |
| Rv_insert_galU | GGTTAAGGCCGTTTGGCATTGCTAGCCCATGGTTATTACTTCTTAATGCCCATCTC |
| Rv_insert_UgpA | GGTTAAGGCCGTTTGGCATTGCTAGCCCATGGTTATCACACCCAATCACCGGGCTCGATG |
| Fw_pCX_backbone | GAAGGCGGCGGTGGAATCGAAATC |
| Rv_pCX_backbone | CGTGAGTTTTCGTTCCACTGAGCGTCAGAC |
| Fw_L4_BaSP | TCACGAGATTTCGATTCCACCGCCGCCTTCAGACGAATTACTTATCTGGCAGGAGTATC |
| Rv_BasP_L5 | GTCTGACGCTCAGTGGAACGAAAACTCACGCTGAAGGCGCTCCTCATACTG |
| Fw_ori_pCX | CGTTCCACTGAGCGTCAGAC |
| Rv_L5_stitch | GCCGTTGCACTAATACAGGGTAATTC |
| Fw_L5_stitch | GAGGGAATTACCCTGTATTAG |
| Rv_L6_end | TTTCTACGGGGTCTGACGCTCAGTGGAACGGTTCCGATGGCGTGCATCAG |
| Rv_L6_stitch | GTAAAGCGGGCGCACCCTCTGAGAATTAAC |
| Fw_L6_stitch | GTTAATTCTCAGAGGGTGCGCCCGCTTTAC |
| Rv_L7_end | TTTCTACGGGGTCTGACGCTCAGTGGAACGCATCTTCTTCGAGTGGTCCCAGAAC |
| Fw_P22_galE | CCGTCGACCTCGAATTCGGAGGAAACAAAGATGAGAGTTCTGGTTACCGGTGGTAG |
| Rv_galE_T4 | GAACGGCAATGCTAGCCCATGGTTATTAATCGGGATATCCCTGTGGATGG |
| Fw_P22_galE2 | CCGTCGACCTCGAATTCGGAGGAAACAAAGATGACAGTTCTCGTTACCGGTGGGTG |
| Rv_galE2_T4 | GAACGGCAATGCTAGCCCATGGTTATCAGCCGACCTGCTTCCAGGTG |
| Rv_bb_general | CTTTGTTTCCTCCGAATTCGAGGTC |
| Fw_L4_stitch | CGATGGTCAAGCGTGGTCTG |
| Rv_L4_stitch | AGACTTCAGGCAGACCACGC |
| Fw_L5-stitch_CLIVA | CGCGGA*CATGAT*TTTGATTG |
| Rv_L6-stitch_CLIVA | CGCTGC*GGCAAT*CTTATGG |
| Fw_L6-stitch_CLIVA | ATTGCC*GCAGCG*CTTTATTG |
| Rv_L5-stitch_CLIVA | ATCATG*TCCGCG*CCAGTTTC |
| Rv_SuSy_T6 | CCGTTTGGCATTGCTAGCCCATGGTTATTATTCAACTGCCAGCGGAACC |
| Rv_L6_P22 | ATTTATAAATGAAGCGGCCGCCCGGGATGTTCCGATGGCGTGCATCAG |
| Rv_L7_ori | TTTCTACGGGGTCTGACGCTCAGTGGAACGCATCTTCTTCGAGTGGTCCCAGAAC |
| Rv_L4_P22 | ATTTATAAATGAAGCGGCCGCCCGGGATAGACTTCAGGCAGACCACGCTTGAC |
| Rv_galK_T4 | GGAACGGCAATGCTAGCCCATGGTTATCAGCACTGTCCTGCTCCTTG |
