# Supplementary material for: Metabolic engineering of Escherichia coli into a versatile glycosylation platform: production of bio-active quercetin glycosides
Source: Microb Cell Fact. 2015 Sep 16;14:138. doi: 10.1186/s12934-015-0326-1 (PMC4573293; doi:10.1186/s12934-015-0326-1)
Supplement: Supplementary file 3 — Additional file 3: Figure S1. Cloning strategy for plasmids of the galactosylation and rhamnosylation platform [file 12934_2015_326_MOESM3_ESM.docx]

**Cloning strategy for plasmids of the galactosylation platform**

**Figure S1A**

**The expression plasmid pBaSP/VvGT2/UgpA [**[**1**](#_ENREF_1)**] was used as a PCR template for the generation of the backbone, while pF3GT was used as PCR template for the insert. The *f3gt* sequence [Genbank: AF165148] from *Petunia hybrid* was codon optimized and synthesized by GeneArt® (Life Technologies). Assembly by CLIVA [**[**2**](#_ENREF_2)**] generated the intermediate plasmid pBaSP/F3GT/UgpA. Genes are under control of constitutive promoter P22 [**[**3**](#_ENREF_3)**] and flanked by homologous linkers L (200 bp) for easy assembly.**

**Figure S1B**

**The *galE* [Genbank: JW0742] and *galE2* [Genbank: KJ543703] sequences were amplified from the genomic DNA of *E. coli* and *Bifidobacterium* *bifidum*, respectively, and inserted in a pUC57 backbone under control of a constitutive promoter P22. This resulted in plasmids pGalE and pGalE2, which were used for the generation of P22-GalE inserts. pBaSP/F3GT/UgpA was used for the amplification of the F3GT/UgpA backbone. Gibson assembly of the GalE or GalE2 inserts with this backbone resulted in the final galactosylation plasmids pGalE/F3GT/UgpA and pGalE2/F3GT/UgpA, respectively.**

**Cloning strategy for plasmid of the rhamnosylation platform**

**Figure S1C**

**Plasmids pRhaGT and pMUM4 were synthesized by GeneArt® (Life Technologies). The *MUM4* sequence[Genbank: AT1G53500] and *RhaGT* sequence [Genbank: AF360160] from *A. thaliana* were codon optimized for *E. coli* .Similarly, MUM4 and RhaGT were introduced using a 3-pieces Gibson assembly, resulting in the final rhamnosylation plasmid pMUM4/RhaGT/UgpA.**

**S1D: Sequences of codon optimized genes**

>RhaGT_optim

ATGACCAAATTTAGCGAACCGATTCGTGATAGCCATGTTGCAGTTCTGGCATTTTTTCCGGTTGGTGCACATGCAGGTCCGCTGCTGGCAGTTACCCGTCGTCTGGCAGCAGCAAGCCCGAGCACCATTTTTAGCTTTTTTAACACCGCACGTAGCAATGCAAGCCTGTTTAGCAGCGATCATCCGGAAAACATTAAAGTGCATGATGTTAGTGATGGTGTTCCGGAAGGCACCATGCTGGGTAATCCGCTGGAAATGGTTGAACTGTTTCTGGAAGCAGCACCGCGTATTTTTCGTAGCGAAATTGCAGCAGCAGAAATTGAAGTTGGTAAAAAAGTTACCTGCATGCTGACCGATGCCTTTTTTTGGTTTGCAGCAGATATTGCAGCCGAACTGAATGCAACCTGGGTTGCCTTTTGGGCAGGCGGTGCAAATAGCCTGTGTGCACATCTGTATACCGATCTGATTCGTGAAACCATTGGTCTGAAAGATGTTAGCATGGAAGAAACCCTGGGTTTTATTCCGGGTATGGAAAATTATCGCGTGAAAGATATTCCGGAAGAAGTGGTTTTTGAAGATCTGGATAGCGTTTTTCCGAAAGCACTGTATCAGATGAGCCTGGCACTGCCTCGTGCAAGCGCAGTGTTTATTAGCAGCTTTGAGGAACTGGAACCGACCCTGAATTATAACCTGCGTAGCAAACTGAAACGCTTCCTGAATATTGCACCGCTGACCCTGCTGAGCAGCACCAGCGAAAAAGAAATGCGTGATCCGCATGGTTGTTTTGCATGGATGGGTAAACGTAGCGCAGCAAGCGTTGCATATATTAGCTTTGGCACCGTTATGGAACCGCCACCTGAAGAACTGGTTGCAATTGCCCAGGGTCTGGAAAGCAGTAAAGTTCCGTTTGTTTGGAGCCTGAAAGAAAAAAACATGGTGCATCTGCCGAAAGGTTTTCTGGATCGTACCCGTGAACAGGGTATTGTTGTTCCGTGGGCACCTCAGGTGGAACTGCTGAAACATGAAGCAATGGGTGTTAATGTTACCCATTGTGGTTGGAATAGCGTGCTGGAAAGCGTTTCTGCCGGTGTTCCGATGATTGGTCGTCCGATTCTGGCAGATAATCGTCTGAATGGTCGTGCAGTTGAAGTTGTTTGGAAAGTTGGTGTGATGATGGATAATGGCGTGTTTACCAAAGAAGGCTTTGAGAAATGCCTGAACGATGTGTTTGTTCACGATGATGGTAAAACCATGAAAGCCAATGCCAAAAAACTGAAAGAGAAACTGCAAGAGGACTTCAGCATGAAAGGTAGCAGCCTGGAAAATTTCAAAATCCTGCTGGATGAAATCGTGAAAGTGTAA

>MUM4_optim

ATGGATGACACCACCTATAAACCGAAAAACATTCTGATTACCGGTGCCGCAGGTTTTATTGCAAGCCATGTTGCAAATCGTCTGATTCGTAATTATCCGGACTATAAAATCGTGGTGCTGGATAAACTGGATTATTGCAGCGATCTGAAAAATCTGGATCCGAGCTTTAGCAGCCCGAATTTCAAATTTGTGAAAGGCGATATTGCCTCCGATGATCTGGTTAATTATCTGCTGATCACCGAAAACATCGATACCATTATGCATTTTGCAGCACAGACCCATGTGGATAATAGCTTTGGTAACAGCTTTGAATTTACCAAAAACAACATCTATGGCACCCATGTTCTGCTGGAAGCATGTAAAGTTACCGGTCAGATTCGTCGTTTTATTCATGTTAGCACCGATGAAGTTTATGGCGAAACGGATGAAGATGCAGCAGTTGGTAATCATGAAGCAAGCCAGCTGCTGCCGACCAATCCGTATAGCGCAACCAAAGCCGGTGCAGAAATGCTGGTTATGGCATATGGTCGTAGCTATGGTCTGCCGGTTATTACCACCCGTGGCAATAATGTTTATGGTCCGAATCAGTTCCCGGAAAAAATGATCCCGAAATTCATTCTGCTGGCAATGAGCGGTAAACCGCTGCCGATTCATGGTGATGGTAGCAATGTGCGTAGCTATCTGTATTGTGAAGATGTTGCAGAAGCCTTTGAAGTCGTTCTGCATAAAGGTGAAATTGGCCATGTTTATAATGTGGGCACCAAACGTGAACGTCGTGTTATTGATGTGGCACGTGATATTTGTAAACTGTTTGGTAAAGATCCGGAAAGCAGCATTCAGTTTGTGGAAAATCGTCCGTTTAATGATCAGCGCTATTTTCTGGATGACCAGAAACTGAAAAAACTGGGTTGGCAAGAACGTACCAATTGGGAAGATGGTCTGAAAAAAACCATGGATTGGTATACCCAGAATCCGGAATGGTGGGGTGATGTTAGCGGTGCACTGCTGCCGCATCCGCGTATGCTGATGATGCCTGGTGGTCGTCTGAGTGATGGTTCAAGCGAAAAAAAAGATGTTAGCAGCAATACCGTTCAGACCTTTACCGTTGTTACCCCGAAAAATGGTGATAGCGGTGATAAAGCAAGCCTGAAATTTCTGATTTATGGCAAAACCGGTTGGCTGGGTGGTCTGCTGGGCAAACTGTGTGAAAAACAGGGTATTACCTATGAGTATGGTAAAGGCCGTCTGGAAGATCGTGCAAGCCTGGTTGCAGATATTCGTAGCATTAAACCGACCCATGTTTTTAACGCAGCAGGCCTGACCGGTCGTCCGAATGTTGATTGGTGTGAAAGCCATAAACCGGAAACAATTCGTGTTAATGTTGCAGGCACCCTGACCCTGGCAGATGTTTGTCGTGAAAATGATCTGCTGATGATGAATTTTGCCACCGGCTGCATTTTTGAATATGATGCAACCCATCCGGAAGGTAGCGGTATTGGTTTTAAAGAAGAAGATAAACCTAACTTTTTTGGCAGCTTCTATAGCAAAACCAAAGCAATGGTTGAAGAACTGCTGCGCGAATTTGATAATGTTTGTACCCTGCGTGTTCGTATGCCGATTAGCAGTGATCTGAATAACCCTCGTAACTTCATTACCAAAATCAGCCGCTATAACAAAGTGGTGGATATTCCGAATAGCATGACCGTACTGGATGAACTGCTGCCGATTAGTATTGAAATGGCAAAACGTAATCTGCGTGGCATTTGGAACTTTACCAATCCGGGTGTTGTTAGCCATAACGAAATTCTGGAAATGTACAAAAACTACATCGAACCGGGTTTTAAATGGTCCAATTTTACCGTGGAAGAACAGGCCAAAGTTATTGTTGCAGCACGTAGCAATAATGAAATGGATGGTAGTAAACTGAGCAAAGAATTTCCTGAAATGCTGAGCATTAAAGAAAGCCTGCTGAAATATGTGTTCGAACCGAATAAACGCACCTAA

>F3GT_optim

ATGAGCAATTATCATGTTGCCGTTCTGGCATTTCCGTTTGCAACCCATGCAGGTCTGCTGCTGGGTCTGGTTCAGCGTCTGGCAAATGCACTGCCGAATGTTACCTTTACCTTTTTCAATACCAGCAAAAGCAACAGCAGCCTGTTTACCACACCGCATGATAATAACATCAAACCGTTTAACATTAGTGATGGTGTGCCGGAAGGTTATGTTGTTGGTAAAGGTGGCATTGAAGCACTGATTGGTCTGTTTTTCAAAAGCGCCAAAGAAAACATTCAGAATGCAATGGCAGCAGCAGTTGAAGAAAGCGGCAAAAAAATCACCTGTGTTATGGCAGATGCCTTTATGTGGTTTAGCGGTGAAATTGCAGAAGAACTGAGCGTTGGTTGGATTCCGCTGTGGACCAGCGCAGCAGGTAGCCTGAGCGTTCATGTTTATACCGATCTGATTCGTGAAAATGTTGAAGCACAGGGTATTGCAGGTCGTGAAGATGAAATTCTGACCTTTATTCCGGGTTTTGCAGAACTGCGTCTGGGTAGCCTGCCGAGCGGTGTTGTGAGCGGTGATCTGGAAAGCCCGTTTAGCGTTATGCTGCATAAAATGGGTAAAACCATTGGTAAAGCAACCGCACTGCCGGTTAATAGCTTTGAAGAACTGGATCCGCCTATTGTGGAAGATCTGAAAAGCAAATTTAACAACTTCCTGAACGTGGGTCCGTTTAATCTGACCACTCCGCCTCCGAGCGCAAATATTACCGATGAATATGGTTGTATTGCCTGGCTGGATAAACAAGAACCGGGTAGCGTTGCATATATTGGTTTTGGCACCGTTGCAACACCGCCACCGAATGAACTGAAAGCAATGGCCGAAGCCCTGGAAGAAAGCAAAACCCCGTTTCTGTGGTCACTGAAAGACCTGTTTAAAAGCTTCTTTCCGGAAGGCTTTCTGGAACGTACCAGCGAATATGGTAAAATTGTTAGCTGGGCACCGCAGGTTCAGGTTCTGAGCCATGGTAGCGTGGGTGTTTTTATCAATCATTGTGGTTGGAATAGCGTGCTGGAAAGCATTGCAGCCGGTGTTCCGGTTATTTGTCGTCCGTTTTTTGGTGATCATCAGCTGAATGCATGGATGGTTGAAAAAGTGTGGAAAATCGGCGTGAAAATTGAAGGTGGTGTGTTTACCAAAGATGGCACCATGCTGGCACTGGATCTGGTGCTGAGCAAAGATAAACGTAATACCGAACTGAAACAGCAGATCGGCATGTATAAAGAACTGGCACTGAATGCAGTTGGTCCGAGCGGTAGCAGCGCAGAAAATTTCAAAAAACTGGTGGATATTATCACCAGCTGCAATTAA

1. De Bruyn F, De Paepe B, Maertens J, Beauprez J, De Cocker P, Mincke S et al. Development of an in vivo glucosylation platform by coupling production to growth: Production of phenolic glucosides by a glycosyltransferase of Vitis vinifera. Biotechnol Bioeng. 2015. doi:10.1002/bit.25570.

2. Zou R, Zhou K, Stephanopoulos G, Too HP. Combinatorial engineering of 1-deoxy-D-xylulose 5-phosphate pathway using cross-lapping in vitro assembly (CLIVA) method. PloS one. 2013;8(11):e79557. doi:10.1371/journal.pone.0079557.

3. De Mey M, Maertens J, Lequeux GJ, Soetaert WK, Vandamme EJ. Construction and model-based analysis of a promoter library for *E. coli*: an indispensable tool for metabolic engineering. BMC Biotechnol. 2007;7(34). doi:Artn 34

Doi 10.1186/1472-6750-7-34.
